# Feature tracking strain is a promising functional analysis technique in single ventricle patients

**DOI:** 10.1186/1532-429X-15-S1-P297

**Published:** 2013-01-30

**Authors:** Shafkat Anwar, Matthew A Harris, Marc S Keller, Mark A Fogel, Kevin K Whitehead

**Affiliations:** 1 Cardiology, Children's Hospital of Philadelphia, Philadelphia, PA, USA

## Background

Feature-tracking Strain (FT S) is a new technique that performs myocardial Strain evaluation on Steady State Free Precession (SSFP) cines without the need for myocardial tagging. As a pilot study to determine whether FT S may be useful in single ventricle patients, we applied this analysis to patients with hypoplastic left heart syndrome (HLHS) and tricuspid atresia (TA).

## Methods

**Figure 1:**
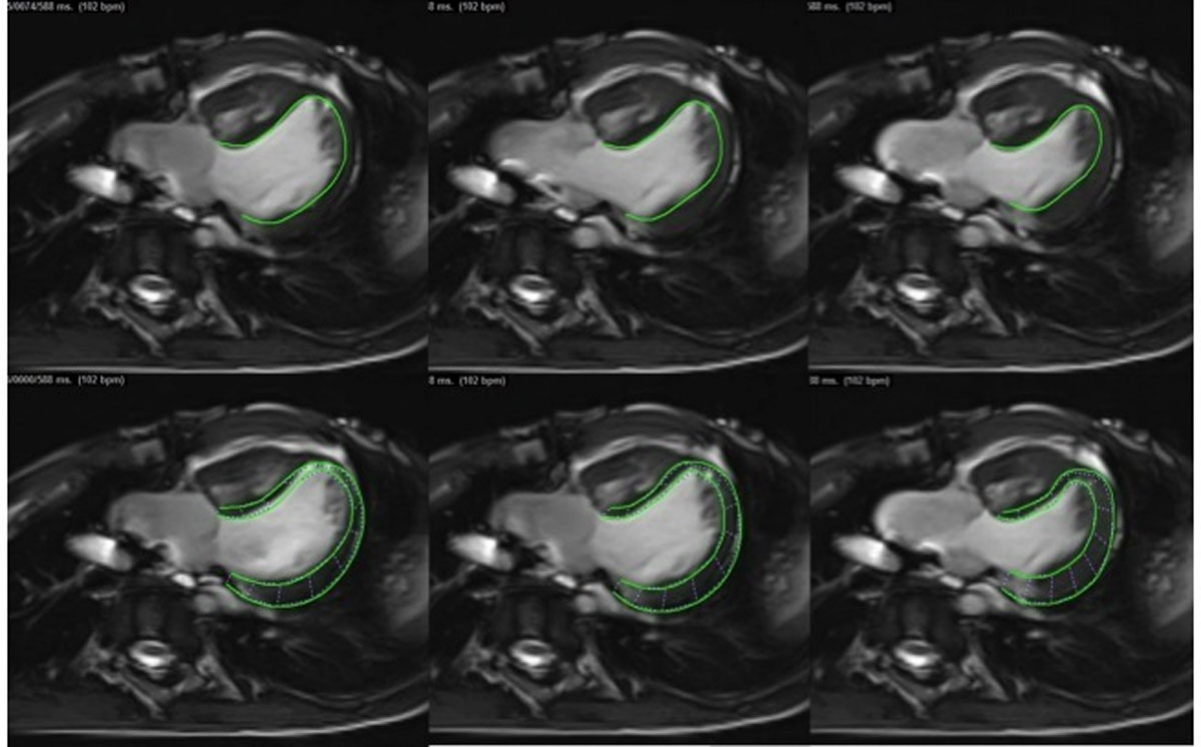
Feature tracking Strain analysis of SSFP cines from end-diastole (left) to end-systole (right) with endocardium tracking protocol (upper panel) and endocardium to epicardium tracking protocol (lower panel)

We retrospectively reviewed 22 patients with a history of Fontan palliation (median age 8.2, range 2 - 27.5 years) who had cardiac MRIs with a Siemens 1.5 Tesla Avanto (Siemens Medical Systems) scanner, 11 with TA, 11 with HLHS. All had steady-state free precession (SSFP) sequences of 4-chamber (4ch) cine and short axis (SAx) cine stacks from cardiac base to apex. Ejection fraction (EF) was calculated from the SAx stack on a Leonardo workstation (Siemens, Inc.). FT S analysis of the 4ch cine was performed off-line with TomTec 2D Cardiac Performance Analysis (version 1.0, TomTec Imaging Systems) following two tracking protocols: endocardial trace only (Endo), epicardial to endocardial trace (Endo-Epi) (Figure 1). Pearson correlation was used to test relationship between absolute global longitudinal Strain (GLSabs) and EF. Differences in GLS between tracking methods were tested with Bland Altman analysis.

## Results

**Figure 2:**
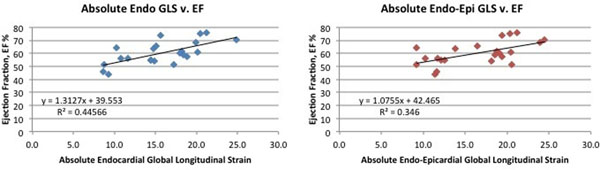
Correlation between ejection fraction and absolute global longitudinal Strain

Mean EF was 60.2%, 95% CI 42.6% to 77.8%. Mean GLS was -15.7 (95% CI -6.8 to -24.7) by Endo., and -16.5 (95% CI -6.9 to -26.1) by Endo-Epi tracking methods. There was fair correlation (Figure 2) between GLSabs and EF using the Endo method (Pearson r = 0.67, p < 0.0001), with poorer correlation using the combined Endo-Epi method (Pearson r = 0.59, p = 0.004). Bland Altman showed Endo-Epi tracking produced slightly higher GLS values compared to tracking Endo alone, mean Endo-Epi GLS > mean Endo GLS of 0.8, with a wide distribution in difference values, 95% CI 2.9 to -4.5. Morphologic ventricle type (single LV vs single RV) did not make a difference in GLS to EF correlations when compared to the mixed cohort. Mean GLS for single LVs was somewhat higher than single RVs, but this difference did not reach statistical significance.

## Conclusions

There is fair correlation between GLS and EF in single ventricle patients with feature-tracking Strain analysis tracking the ventricular endocardium. Validation in a larger sample is warranted. Comparison of FT S with tagged harmonic phase MRI in single ventricle patients may further validate this promising technique in defining regional and global strain.

## Funding

Dr Kevin K.Whitehead was supported in part by NIH K23 Grant HL089647 from the National Heart, Lung and Blood Institute.

Dr. Mark Fogel was supported in part by NIH - R01HL098252-01, "Understanding mechanisms of Fontan failure and key predictors for patient outcome."

